# Effect of Different Types of Strength Training on Swimming Performance in Competitive Swimmers: A Systematic Review

**DOI:** 10.1186/s40798-022-00410-5

**Published:** 2022-01-31

**Authors:** Line Fone, Roland van den Tillaar

**Affiliations:** Department of Sport Sciences and Physical Education, Nord University, Levanger, Norway

**Keywords:** Swimming, Strength training, Effect size, Specificity

## Abstract

**Background:**

Strength training is widely used in swimming for improvement in performance. There are several ways to embark on strength training, which to different degrees follows the principle of specificity. There are disagreements in the literature on which training methods lead to the greatest performance improvements and to what degree resistance training must be specific to swimming to transfer to swimming performance.

**Objective:**

The study was undertaken to examine (1) how different approaches to strength training for competitive swimmers can improve swimming performance and (2) which form of strength training resulted in the largest improvement in swimming performance.

**Methods:**

A systematic review of the literature was undertaken using the following databases: PubMed, SPORTDiscus and Scopus. Studies were eligible if they met the following criteria: (1) a training intervention lasting longer than 3 weeks that investigates the effects strength training has on swimming performance, (2) involves youth or older experienced swimmers, (3) involves in-water specific resistance training, dry-land swim-like resistance training or non-specific dry-land strength training and (4) interventions with clear pre- and posttest results stated. Non-English language articles were excluded. Percent change and between-group effect size (ES) were calculated to compare the effects of different training interventions.

**Results:**

A range of studies investigating different strength training methods were examined. The percent change in performance and between-group ES were calculated; 27 studies met the inclusion criteria. The review revealed no clear consensus on which method of strength training was the most beneficial to swimming performance. All methods had intervention groups that increased their swimming performance.

**Conclusions:**

This review shows that swimming differs from other sports as it is performed in water, and this demands a specific way of training. The results show that a combined swimming and strength training regimen seemed to have a better effect on swimming performance than a swim-only approach to training. Based on the principle of specificity and gains in swimming performance, there is not a clear conclusion, as the three main methods of strength training revealed similar gains in swimming performance of 2–2.5%.

## Key Points

- This systematic review highlights the effects of different strength training forms on swimming performance.
- In general, a combined swimming and strength training regimen is more effective than a swim-only approach to training to achieve gains in swimming performance.
- It is not clear whether transfer of strength training follows the principle of specificity.

## Introduction

Swimming as a competitive sport is popular worldwide and has been a part of the Olympic program since the first modern Olympic Games in 1896. Today, competitive swimming includes 16 Olympic pool events from 50 to 1500 m lasting from approximately 21 s to 15 min. Swimming differs from most other sports in several aspects, such as: (1) swimmers are in a prone, horizontal position during performance and training; (2) both arms and legs are used actively for propulsion; (3) water immersion causes pressure on the body and affects breathing; (4) aside from starts and turns, the forces from the athlete are at all times applied to a moving element; and (5) the equipment (e.g. swimming suit and cap) used during swimming has a minimal effect on swimming performance [1]. Nevertheless, swimming performance is determined by physiological, psychological and anatomical factors [2–6]. Barbosa et al. [7] specified that swimming performance depends on energetics, kinematics (the relationship between swim velocity [v], stroke length [SL] and stroke frequency [SF]) and kinetics (a swimmer creates work energy [kinetic energy] by propelling through the water). Loss of energy transfer is caused by inefficient movement, motor control (coordination of multiple segments at the same time to propel the swimmer forward), anthropometrics (e.g., body proportions, wingspan, body length and mass) and strength and conditioning. Many of these factors are hard, if not impossible, to change (e.g., body proportions and wingspan. Others are hard to investigate and measure (e.g., improvements in technique caused by better motor control). Therefore, this review will only discuss the relationship between strength and swimming performance. In these kinds of training interventions, it is easier to control the variables and get an accurate explanation for the changes in swimming performance.

Swimmers need great mechanical power output and muscular strength for good swimming performance [8]. Therefore, the ability to apply force in water is crucial in competitive swimming [9–12]. Upper body strength is essential in swimming for these propulsive forces and thereby swimming velocity [2, 5]. Consequently, coaches and trainers use strength and conditioning programs to increase strength in athletes. Strength and conditioning (S&C) and dry-land training are common practices in swimming with the aim of enhancing swimming performance [7, 13, 14].

Many studies have examined the effects of strength and conditioning training on swimming performance, but the evidence that this form of training is beneficial for performance enhancement is not yet clarified in the literature. Some literature demonstrates a correlation between upper body strength and swimming performance [9, 15–18]. Others have found a weak-moderate or nonsignificant correlation between strength and swimming performance [8, 19, 20]. Barbosa et al. [7] suggested that reasons for a weak relationship between dry-land strength and swimming performance are rooted in transfer issues between dry-land and aquatic-based strength (a lack in specificity). Furthermore, dry-land strength does not relate directly with swimming performance but indirectly through effects that dry-land strength training has on motor control, anthropometrics, biomechanics, etc.

Sadowski et al. [21] showed that the rate of transfer to swimming performance was significantly higher in a group that used a specialized ergometer for specific strength training as compared to that in a group that trained with traditional resistance exercises. Girold et al. [12], on the other hand, found that their traditional strength training group and the group that engaged specific strength training in the pool using resistance bands both gained similarly in swimming performance. Crowley et al. [22] performed a systematic review which explored the transfer of resistance-training modalities to swimming performance, and examined the effects of resistance training on technical aspects of swimming. They only reviewed fourteen studies of which ten were dryland resistance training and four swim-specific resistance-training methods at that time. The review concluded that low-volume, high-velocity/force, swim-specific resistance-training showed a positive transfer to swimming performance. However, the review [22] also identified that there is a lack of high-quality methodological studies at that time. Furthermore, they did not perform a systematic analysis of effect sizes and percentage of change in swimming performance between the studies. Therefore, the present study aims to review exercise training interventions to clarify what kind of strength training is beneficial for athletes to incorporate in their training routines for a gain in swimming performance. The focus of this review is to determine whether general dry-land strength training or swim-specific resistance training has the most transfer to swim performance in experienced competitive swimmers.

## Methods

### Literature Search

**Fig. 1:**
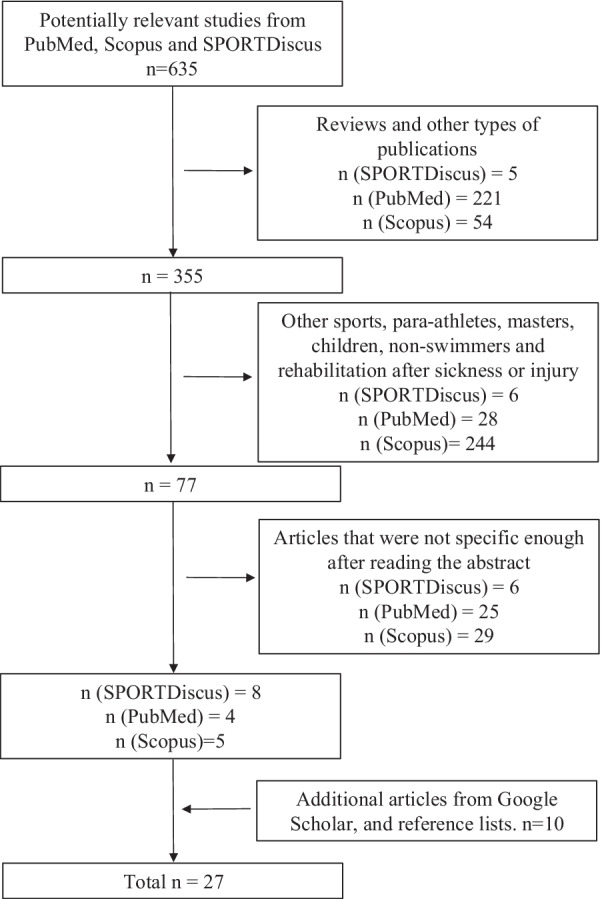
A schematic representation of the searching process to find eligible studies for this review. A PRISMA flowchart was used to illustrate the inclusion and exclusion criteria used in this review

To find eligible literature for this review, an extensive search for exercise training intervention studies designed to improve swimming performance through different forms of strength training was conducted on the 30th of March 2021. The main databases utilized in this research were PubMed, Scopus and SPORTDiscus. In all databases, the main keywords were “swimming performance” and “strength training.” “Swimming” combined with “dry-land strength training,” “specific strength training” and “in-water strength training” were used as secondary searches. “Resistance training” and “weight training” were tried as a substitute for “strength training” in all databases. Complementary searches were done in Google Scholar. When systematic reviews, qualitative reviews and meta-analyses came up in the search that seemed relevant, a thorough screening of their references was conducted alongside a screening of eligible literature bibliographies and cross-references. When articles with a restricted full text online came up in the searches, they were requested and full access to them was gained. Figure 1 shows the complete searching process through a PRISMA flowchart.

### Inclusion and Exclusion Criteria

Only articles written in English were included in this review. Studies published before 1988 were excluded. A thorough screening of titles was conducted. Abstracts and articles written about other sports related to swimming (e.g., water polo, triathlon, open water swimmer and diving) were eliminated. Articles about sick, injured or paraplegic athletes and rehabilitation of patients related to swimming were also excluded. Studies applying supplements or any external manipulative intervention (e.g., wet suits, cold water immersion, electrical stimulation or altitude exposure), studies focus on tapering and recovery, studies surrounding respiratory training and correlation studies (e.g., stroke length and stroke rate; upper body strength and tethered swim force; or sprint performance and dry-land power) fell beyond the aim of this review and were excluded.

To get a relatively coherent pool of subjects, studies with young children, master swimmers and non-swimmers were also eliminated. This review will focus on competitive swimmers above the age of 13 and with a competitive level of at least a regional level. The subjects in this study are both male and female. Thirteen was set as the lowest age due to the uncertainty younger children represent in training interventions. Newer swimming training intervention studies with children have a tendency to report positive effects of the various strength training interventions [23–27], but it is difficult to determine if the swimming performance enhancement or decrement is due to the training interventions or factors such as maturation, physical growth, motivation, improvement in technique, psychological factors or a combination of several of these [28]. A mixture of male and female athletes was necessary to retrieve enough literature for this study, even though it could be argued that this, alongside the relatively wide age span of the participants, will compromise the accuracy of the results. Start and turn studies will not be covered in this review and were, therefore, eliminated from the search process.

To compare the effect of the different strength training interventions on swimming performance, the percentage of change in swimming performance was calculated together with the group effect size (ES) to determine whether the interventions have a real practical effect on the experimental groups compared to the control groups. The between-group ESs were sampled according to Cohen’s d $\frac{Post\, CG-Post EG}{SD\, pooled}$. ESs below 0.2 were defined as trivial effects, 0.2–0.5 small effects, 0.5–0.8 medium effects and 0.8–1.2 large effects. Furthermore, the ES of 1.2–2.0 was defined as a very large effect and ES above 2.0 as a huge effect.

## Results

### General Findings

A total of 27 studies were eligible for the present review. To compare the effect of different methods of strength training on swimming performance, the 27 studies were divided into groups based on the specificity principle. They were constructed from the most specific to swimming to the least specific to swimming. In-water resistance training methods are the most specific, followed by dry-land swim-like resistance training and then the least specific dry-land resistance training methods, such as hypertrophy training, core training and maximal strength training. This categorization makes it possible to investigate if the most specific method to strength train has the largest transfer to swimming and leads to the largest gains in performance, thus following the principle of specificity.

From the 27 identified articles, 10 examined specific in-water resistance training with resistance bands [29–32], hand paddles [33], drag suit or parachute training [34, 35], leg kicking training [36], arms-only training [37] and training with a specialized fixed push-off point (POP) device [38]. Four studies investigated swim-like specific dry-land resistance training [21, 39–41], and 11 studies focused on non-specific dry-land strength training [8, 9, 42–51]. Junior et al. [48] and Girold et al. [12] had two intervention groups and one control group, whereas one intervention group performed specific in-water resistance training and the other group performed non-specific dry-land strength training.

The included studies covered competitive swimming distances of 50 m or 50 yards, 100 m or 100 yards, 200 m and 400 m or 400 yards. Sadowski et al. [21] and Sadowski et al. [41] used 25 m sprints in their research. Most studies investigated the swimming style front crawl, but Mavridis et al. [31] investigated 50 m, 100 m and 200 m in the preferred style of the swimmer (an even distribution in all four swimming styles was applied in the study) and Naczk et al. [40] investigated both the 50 m front crawl and 100 m butterfly.

Most studies used, on average, 19.9 participants (range 10–37), except Mavridis et al. [31] who used 82 participants. The sex distribution was 345 men (66.6%) and 173 women (33.4%), with a total of 518 participants (not including Mavridis et al. [31]). The duration of training interventions ranged from 3 to 16 weeks, with an average of 8 weeks.

### Results for Specific In-Water Resistance Training

Out of 12 studies with a specific in-water resistance intervention, 10 studies reported positive effects after the training intervention. Only Barbosa et al. [33], with a hand paddles intervention, and Dragunas et al. [34], with a drag suit intervention, showed no significant change in performance or stroke parameters pre- and post-intervention. Gourgoulis et al. [35], with a parachute intervention, on the other hand, showed a significant gain in the 50 m, 100 m and 200 m front crawl. Regarding swim performance, Girold et al. [32] reported only the resisted swimming groups showed a significant gain in 100 m performance. This was in line with Mavridis et al. [31] who found gains in 100 m and 200 m performance in the preferred swimming style. Girold et al. [12], with a combined resisted-assisted training group, found significant gain in the 50 m front crawl from pre- to posttest. Junior et al. [48] only showed significant improvements in the 25 m all-out sprint not in the 50 m performance. Kojima et al. [30] found significant gains in 50 m velocities in both the experimental and control groups after the participants followed the same sprint training program with and without resistance bands. Konstantaki and Winter [36] and Konstantaki et al. [37] with their leg kicking and arms-only swimming interventions did not find significant change in 400 m and 400 yards front crawl performance but found gains in submaximal oxygen uptake (VO_2_), peak oxygen uptake (VO_2peak_) and exercise intensity at the ventilatory threshold. Papoti et al. [29], with tethered swimming, showed no significant gain in swim performance. The only significant gain was in peak blood lactate. Lastly, Toussaint and Vervoorn [38] used a MAD system (a system to measure active drag), which is a specialized POP device (fixed push-off points) that the swimmers used during in-water swimming training, to increase resistance in the drag phase of the front crawl stroke. They found a significant gain in the 50 m and 200 m front crawl. Unlike Girold et al. [32], Mavridis et al. [31] and Gourgoulis et al. [35] did not find a performance gain in the 100 m front crawl.

### Specific In-Water Strength Training with Focus on the Arms

The interventions shown in Table 1 are specific in-water training interventions with added resistance on the arms in the form of hand paddles, arms-only swimming or the POP device (a fixed push-off point device in the water) of Toussaint and Vervoorn [38].

**Table 1:** Results for specific in-water resistance training interventions with focus on the arms

Reference	Competition level, sex, N in intervention groups, age (y)	Specific in-water resistance training	Week tr./ tr. per week	Resistance training program for EG and any changes in CG habitual training	Swim performance measured	Other measured effects	Positive effects of training intervention	Findings in swim performance * Significant change from pre- to posttest (p < 0.05)
Barbosa et al. [33]	Regional *n* = 20 10 M, 10 F EG, *n* = 10 21.8 ± 1.9y CG, n = 10 22.4 ± 2.3y	Hand-paddles	4/3	EG: 10 × 10 strokes all-out with hand paddles CG: same as EG, but without paddles	50 m fc	Clean SV, SR, SL, tethered SF and RPE	+ none	50 m (s) EG: **Pre** 31.28 ± 4.78 **Post** 31.28 ± 4.86 CG: **Pre** 32.03 ± 4.27 **Post** 31.93 ± 4.30
Konstantaki et al. [37]	Regional *n* = 15 M EG, *n* = 8 16.0 ± 3.0 y CG, *n* = 7 16.0 ± 3.0 y	Arms-only	6/3	EG: Arms-only training as 20% of the weekly swimming distance CG: normal swim practice	372 m fc	186 m arms only time trial, submax. VO_2_, VO_2peak_, exercise intensity at VT	+ 186 m arms only time trial, submax. VO_2_, VO_2peak_, exercise intensity at VT	372 m (s) EG: **Pre** 319 ± 11.0 **Post** 315 ± 16.0 CG: **Pre** 323 ± 8.0 **Post** 320 ± 10.0
Toussaint and Vervoorn [38]	National *n* = 22 16 M, 6 F EG, *n* = 11 18.40 ± 2.10 y CG, *n* = 11 18.50 ± 3.30 y	POP training device	10 /3	EG: Ex. Training program: 20 × 23 m; 1 length sprinting on the POP, the other swimming back slowly 10 × 23 m kicking 6 × 23 m 3x (1 length sprint on POP, 1 length butterfly) CG: same sprint training as EG but without the POP device	50 m, 100 m, 200 m fc	Max. SF, stroke velocity and stroke power, no. of strokes in 25 m and 50 m	+ 50 and 200 m fc, max. SF, stroke velocity and stroke power, no. of strokes in 25 and 50 m	50 m (s) EG: **Pre** 27.2 ± 1.8 **Post** 26.6 ± 1.8* CG: **Pre** 27.7 ± 1.9 **Post** 27.3 ± 1.9 100 m (s) EG: **Pre** 59.3 ± 3.6 **Post** 57.4 ± 3.8* CG: **Pre** 60.0 ± 4.4 **Post** 58.7 ± 4.6* 200 m (s) EG: **Pre** 129.6 ± 7.3 **Post** 127.3 ± 9.0* CG: **Pre** 130.8 ± 9.1 **Post** 129.9 ± 1.6

Participant’s characteristics, method of resistance training, duration of training intervention and sessions per week, training program, swimming performance measured, positive effects of training intervention, findings in swim performance and whether they are significant (*p* < 0.05). EG = experimental training group and CG is control group

M = male, F = female, n = number, y = years old, VT = ventilatory threshold, VO_2-peak_ = exercise capacity measured of oxygen uptake, carbon dioxide production, and minute ventilation, fc = front crawl, SV = swimming velocity, SR = stroke rate, SL = stroke length, SF = swimming force and RPE = ratings of perceived exertion with Borg’s scale

### Specific In-Water Strength Training with Added Resistance

With this form of in-water strength training, the main goal is to increase the resistance so that the swimmer, in a very specific way, increases overall strength. The resistance band is attached to the swimmer’s waist and secured in the starting block. The swimmer swims out against the band and then maintains his or her position. In Girold et al. [32], there was one group that swam against the resistance and one that used the resistance band in the opposite way and decreased the total resistance. Most of the studies in Table 2 used resistance bands, but Dragunas et al. [34] used a drag suit, which is a swimming suit with added pockets around the waist that retains water and thereby increases the resistive drag force, resulting in the swimmer using more propulsive force to achieve the same result. The third way to increase resistance was to use a parachute [35]. The parachute was attached to the swimmer’s waist, and while the athlete swims, the parachute expands and creates a big surface. In the same way as the drag suit, this forced the swimmer to increase the propulsive force to attain the same velocity as when the swimmer does not use the parachute.

**Table 2:** Results for specific in-water resistance training interventions

Reference	Competition level, sex, N in intervention groups, age (y)	Specific in-water resistance training	Week tr./ tr. per week	Resistance training program for EG and any changes in CG habitual training	Swim performance measured	Other measured effects	Positive effects of training intervention	Findings in swim performance * Significant change from pre- to posttest (p < 0.05)
Dragunas et al. [34]	Regional to national *n* = 18 10 M, 8 F EG, *n* = 9 19.3 ± 0.87 CG, *n* = 9 19.0 ± 1.80	Drag-suit	5/3	EG: Drag-suit Day 1: 3 × 50yd sprint + 200yd easy swim Day 2: 4 × 4 x 25yd sprints + 200yd easy swim Day 3: 16 × 25yd sprints CG: same sprint sets without drag-suit	50 m fc	SR and SL	+ none	50 m (s) EG: **Pre** 29.6 ± 3.1 **Post** 29.5 ± 2.9 CG: **Pre** 29.8 ± 2.15 **Post** 29.3 ± 2.2
Girold et al. [32]	Regional to national *n* = 37 16 M, 21 F EG (RS), *n* = 15 16.5 ± 2.0 EG (AS), *n* = 11 18.0 ± 3.0 CG, *n* = 11 17.0 ± 3.0	Resistance band	3/3	AS: 12 × 25-m crawl sprints RS: 30 s work /30 s rest × 6 crawl sprints CG: 50 m × 6 crawl sprints	100 m fc	SL, SR, elbow flexor and extensor strength, 1st and 2nd 50 m over the 100 m performance	+ SR (both intervention groups), 100 m (RS)	100 m (s) RS: **Pre** 67.43 ± 4.4 **Post** 66.05 ± 4.0* AS: **Pre** 62.46 ± 5.32 **Post** 61.9 ± 4.85 CG: **Pre** 68.15 ± 6.18 **Post** 68.35 ± 5.91
Girold et al. [12]	Regional to national *n* = 14 7 M, 7 F EG, *n* = 7 16.5 ± 2.5 CG, *n* = 7 16.5 ± 1.5	Resistance band	12/2	RAS: 3 reps × 2 × 3. Resisted one way and assisted the other ca. 45 min CG: 45 min aerobic cycling	50 m fc	SL, SR, SD, elbow flexor and extensor strength	+ 50 m, SD, elbow extensor strength	50 m (s) RAS: **Pre** 30.94 ± 1.59 **Post** 30.00 ± 2.00* CG: **Pre** 31.35 ± 2.30 **Post** 31.11 ± 1.89
Gourgoulis et al. [35]	Regional *n* = 12 F EG, *n* = 6 13.08 ± 0.9 CG, *n* = 6 13.08 ± 0.9	Parachute	11/4	EG: Parachute Day 1 and 3: 3 × 6 × 15-m all-out Day 2 and 4: 2 × 4 × 25-m all-out CG: same as EG but without parachute	50 m, 100 m, 200 m fc	50, 100, 200 m front crawl with pull buoy, mean SV, SL, SR, total stroke duration, duration of propulsive and non-propulsive phase	+ 50, 100, 200 m front crawl with and without pull buoy, mean swimming velocity	50 m (s) EG: **Pre** 35.92 ± 1.96 **Post** 34.77 ± 2.13* CG: **Pre** 35.67 ± 3.50 **Post** 35.60 ± 3.04 100 m (s) EG: **Pre** 77.73 ± 5.25 **Post** 73.75 ± 5.21* CG: **Pre** 78.00 ± 7.46 **Post** 77.10 ± 8.24 200 m (s) EG: **Pre** 172.00 ± 12.98 **Post** 159.17 ± 10.68* CG: **Pre** 171.17 ± 14.47 **Post** 170.17 ± 13.75
Junior et al. [48]	National *n* = 14 M EG, *n* = 7 15–16 CG, *n* = 7 15–16	Resistance band	8/2	RS: 3 × 2 series of 30 s resisted swim + 10 s + 2 min rest CG: swim only	50 m fc	RPE	+ 25 m front crawl	25 m (s) EG: **Pre** 13.10 ± 0.65 **Post** 12.70 ± 0.46* CG: **Pre** 13.15 ± 0.54 **Post** 13.13 ± 0.47 50 m (s) EG: **Pre** 27.50 ± 0.75 **Post** 27.13 ± 0.72 CG: **Pre** 27.55 ± 0.54 **Post** 27.51 ± 0.92
Kojima et al. [30]	Regional *n* = 18 9 M, 9 F EG, *n* = 9 13.5 ± 1.0 CG, *n* = 9 13.5 ± 1.4	Resistance band	10/2	RS: 10 × 10-m resisted sprints CG: 10 × 15-m non-resisted sprints	50 m fc	Peak power, peak power/ stroke, 13.7 m front crawl	+ peak power, peak power per stroke, 13.7 and 50 m fc for both groups	50 m (s) EG: **Pre** 31.85 ± 0.15 **Post** 30.8 ± 4.2* CG: **Pre** 32.47 ± 0.11 **Post** 31.25 ± 1.8*
Mavridis et al. [31]	National *n* = 82 (–-) EG, *n* = 53 14.7 ± 1.5 CG, *n* = 29 15.0 ± 1.5	Resistance band	12/3	RS: 2 × 50 m at 70% intensity 4 × 25 m at max intensity with resistance CG: same as RS but without resistance	50 m, 100 m, 200 m in preferred stroke	10 m test with and without resistance	+ 100 and 200 m in best stroke, 10 m test with and without resistance	50 m (s) EG: **Pre** 33.74 ± 3.85 **Post** 32.55 ± 3.49* CG: **Pre** 33.63 ± 3.87 **Post** 32.97 ± 3.55* 100 m (s) EG: **Pre** 73.13 ± 8.66 **Post** 70.83 ± 7.86* CG: **Pre** 72.91 ± 8.71 **Post** 71.83 ± 7.85 200 m (s) EG: **Pre** 159.59 ± 18.34* **Post** 154.5 ± 17.39 CG: **Pre** 159.19 ± 18.34 **Post** 157.66 ± 17.43
Papoti et al. [29]	National *n* = 21 12 M, 9 F EG, *n* = 10 16.0 ± 2.1 CG, *n* = 11 16.0 ± 2.1	Resistance band	7/5	EG: 50% of main series performed with tethered swimming CG: swim only	100 m, 200 m and 400 m fc	Anaerobic threshold, SR at anaerobic threshold, peak force, peak blood lactate	+ peak blood lactate	100 m (s) EG: **Pre** 65.79 ± 7.01 **Post** 66.67 ± 7.20 CG **Pre** 65.79 ± 7.45 **Post** 67.11 ± 7.76 200 m (s) EG: **Pre** 151.52 ± 11.54 **Post** 148.15 ± 14.40 CG: **Pre** 148.15 ± 17.81 **Post** 148.15 ± 15.53 400 m (s) EG: **Pre** 320.00 ± 31.01 **Post** 320.00 ± 28.38 CG: **Pre** 320.00 ± 28.38 **Post** 322.58 ± 23.54

Participant’s characteristics, method of resistance training, duration of training intervention and sessions per week, training program, swimming performance measured, positive effects of training intervention, findings in swim performance and whether they are significant (*p* < 0.05). EG = experimental training group and CG is control group

M = male, F = female, n = number, y = years, yd = yards, fc = front crawl, RS = resistance trained group, AS = assisted trained group, SR = stroke rate, SL = stroke length, SD = stroke depth, SV = swimming velocity, RPE = ratings of perceived exertion with Borg’s scale

### Specific In-water Strength Training with Focus on the Legs

Only Konstantaki and Winter [36] focused on increasing leg strength and performed a leg kicking study (Table 3).

**Table 3:** Results for specific in-water leg-kicking training interventions

Reference	Competition level, sex, N intervention groups, age (y)	Specific in-water resistance training	Week tr./ tr. per week	Resistance training program for EG and any changes in CG habitual training	Swim performance measured	Other measured effects	Positive effects of training intervention	Findings in swim performance * Significant change from pre- to posttest (p < 0.05)
Konstantaki and Winter [36]	Regional *n* = 15 M EG, *n* = 8 16.0 ± 5.0 CG, *n* = 7 16.0 ± 5.0	Leg-kicking	6/3	EG: Leg-kicking training as 20% of the weekly swimming distance CG: normal swim practice	400 m fc	200 m leg-kicking time trial, submax. VO_2_, VO_2peak_, exercise intensity at VT	+ 200 m leg-kicking time trial, submax. VO_2_, VO_2peak_, exercise intensity at VT	400 m (s) EG: **Pre** 309 ± 25.0 **Post** 307 ± 20.0 CG: **Pre** 313 ± 19.0 **Post** 311 ± 21.0

Participant’s characteristics, method of resistance training, duration of training intervention and sessions per week, training program, swimming performance measured, positive effects of training intervention, findings in swim performance and whether they are significant (p < 0.05). EG = experimental training group and CG is control group

M = male, F = female, n = number, y = years old, fc = front crawl, VT = ventilatory threshed

### Results from Specific Dry-land Swim-like Resistance Training

A swim bench is a way to perform specific resistance training out of the pool and is suggested to reproduce some elements of in-water swimming [16, 39]. However, it cannot reproduce the aquatic feeling, which is specific to swimming and is an important component for a swimmer to master in regard to technique and swimming performance. When the swimmer uses the swim bench, he or she lies prone on a sliding bench with a slight incline, arms outstretched over his or her head and hands secured in hand paddles. The swimmer then pulls along the sliding bench and, therefore, mimics the kinematics of front crawl swimming. Sadowski et al. [21] and Sadowski et al. [41] used an ergometer like the swim bench. The ergometer was fastened to the end of the pool. When using the ergometer, the swimmer lies prone on a bench, similar to the position when performing the front crawl, while holding handles connected to a rotary head with blades located in the pool. When the swimmer uses the ergometer, it mimics the underwater phase of the front crawl stroke.

### Results for Non-specific Dry-land Resistance Training

For non-specific dry-land resistance training, there was a large variance in the type of training undertaken by the athletes, what effects were measured, and the reported results of various interventions. Tanaka et al. [47] was the only study in this subgroup of training interventions that reported no positive effects after the training intervention, but Tanaka and colleagues were not alone in the lack of positive gains in swimming performance. Sawdon-Bea and Benson [45] and Schumann et al. [42] did not find significant changes in swimming performance. Junior et al. [48] found significant improvement in a separate 25 m all-out sprint but not in the 50 m front crawl performance. Trappe and Pearson [8] recorded a gain in swimming performance in both groups. In the experimental only group, they found a gain in maximal sprint swimming and maximal arm power in one of three methods utilizing the swim bench. In studies that reported gains in swimming performance, there was disagreement between studies as to which swimming distances were affected. Aspenes et al. [9] reported only significant improvements in the 400 m front crawl. Several studies reported improvements in the 50 m front crawl [12, 43, 44, 49, 51], while Lopes et al. [50] reported gains in both 50 m and 100 m performances. Potdevin et al. [46] reported improvements in 50 m and 400 m velocities.

### Non-specific Dry-land Core Training

This form of training concentrates on increasing strength in the core muscles on the basis that a stronger core is beneficial to overcome the unstable and dynamic nature of the water and is necessary to produce and transfer force between the trunk and upper and lower extremities [52]. Swimming differs from other ground-based sports in that the core becomes the reference point for all movements [52]. The core muscles in these studies include the hip flexors, pelvis, trunk and shoulders.

### Non-specific Dry-land Hypertrophy Training

Hypertrophy training is a training method to increase muscle mass, thereby increasing muscle strength. When using this training method, the athletes often train at 60–80% of 1RM and 6–15 repetitions for 3–5 sets. Junior et al. [48] and Lopes et al. [50] used a full-body training program, while Tanaka et al. [47] and Trappe and Pearson [8] utilized programs that were designed to increase strength in the upper body.

### Non-specific Dry-land Maximal Strength Training

In maximal strength training, the athletes train with > 80% of 1RM with 1–6 repetitions for 3–5 sets, and the goal is to increase strength. Swimming is dependent on power and muscle strength [15–17, 47], with the latter identified as a major component for success in swimming [8]. Strass [43] found that maximal strength training can change the rate of force development and maximal force. The gain in maximal force is influenced primarily by hypertrophy, while the explosive maximal force productions are affected by neural activation and are an important component of the underwater arm movement in sprint swimming.

### Non-specific Dry-land Plyometric Training

Plyometric training is a way to train to enhance explosive strength. The improvement in strength originates from optimizing the stretch–shortening cycle, which occurs when the active muscle switches from rapid eccentric muscle action (deceleration) to rapid concentric muscle action (acceleration), therefore improving muscle function, coordination and the direction of the resultant force [53]. Normally explosive dry-land training in swimming is related to the performance of starts and turns [53, 54], but Potdevin et al. [46] performed a study to see whether plyometric training influenced swimming velocity in the 50 m and 400 m front crawl.

### Combined Strength and Endurance Training

Only one study [9] in this review performed a combined endurance and strength training intervention. The endurance component of the intervention consisted of 4 × 4 min high-intensity swimming at 90–95% of the swimmer’s maximal heart rate. The strength part of the training intervention consisted of maximal strength training on the latissimus dorsi, with maximal force in the concentric part of the movement and a slow eccentric phase [9].

### Percent Change and Effect Sizes in Swimming Performance

**Fig. 2:**
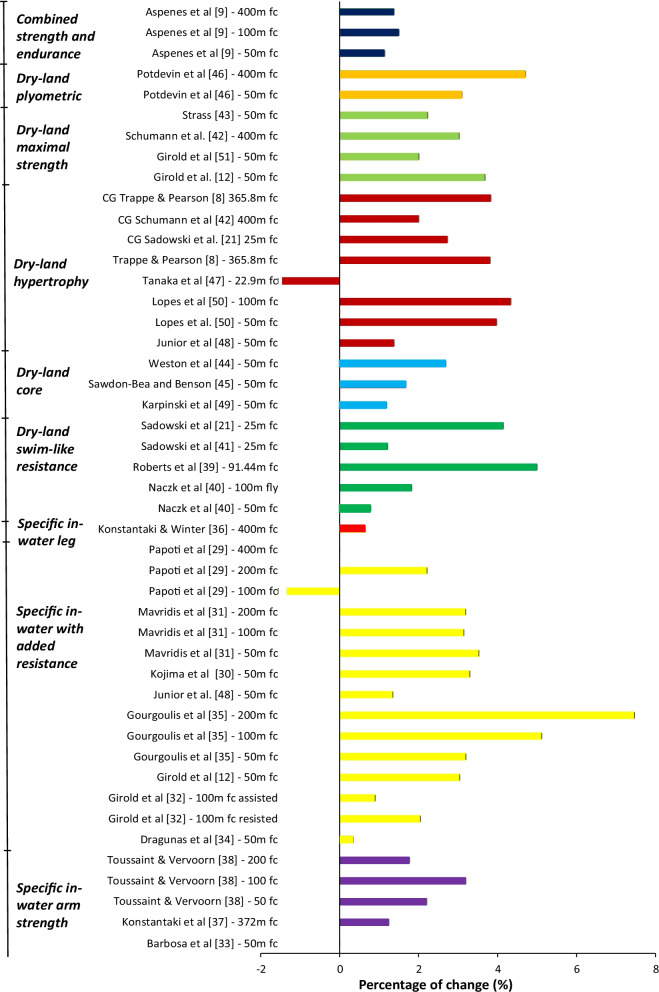
Percent change in swimming performance (s) after a training intervention

In Fig. 2, the percent changes in performance for the experimental groups are presented to compare the effects of different training interventions. Several of the interventions measured different swimming distances and are, therefore, represented individually. Girold et al. [32] had two experimental groups, one resisted and one assisted training group, so they are also represented individually. The results varied from a 7.5% positive response [35] to a negative response of 1.5% [47]. The only other negative response was Papoti et al. [29] in the 100 m front crawl (1.3%). Two experimental groups showed no percent change in swimming in the 400 m front crawl and 50 m front crawl performance [29, 33]. The rest showed positive effects of their training interventions. The gains in performance were mostly in the range of 1% to 3% (Tables 4, 5, 6, 7, 8, 9).

**Table 4:** Results for specific dry-land swim-like resistance training

Reference	Competition level, sex, N in intervention groups, age(y)	Dry-land swim-like resistance training	Week tr./ tr. per week	Resistance training program for EG and any changes in the CG habitual training	Performance measured	Other measured effects	Positive effects of training intervention	Findings in swim performance * Significant change from pre- to posttest (p < 0.05)
Naczk et al. [40]	National *n* = 14 10 M, 4 F EG, *n* = 7 15.8 ± 0.4 CG, *n* = 7 15.8 ± 0.4	Swim bench (Simulating fc and butterfly stroke)	4/3	EG: Swim bench training at max intensity CG: swim only	100 m butterfly, 50 m fc	Bioelectrical impedance analysis (body mass analysis), muscle force and power	+ muscle force and power, 50 m fc and 100 m butterfly	50 m (s) EG: **Pre** 27.74 ± 1.06 **Post** 27.52 ± 0.97* CG: **Pre** 29.38 ± 1.05 **Post** 29.35 ± 0.95 100 m (s) EG: **Pre** 67.94 ± 2.66 **Post** 66.70 ± 2.82* CG: **Pre** 68.34 ± 2.61 **Post** 68.22 ± 2.61
Roberts et al. [39]	National *n* = 16 M EG, *n* = 8 19.1 ± 2.1 CG, *n* = 8 19.1 ± 2.1	Swim bench (simulating fc stroke)	10/3	EG: Biokinetic swim bench 10 s work – 10 s rest × 4 × 3 at max velocity CG: swim only	91.44 m (100yd) fc	Body fat, biokinetic and isokinetic power, anaerobic power, SR, peak lactate	+ none	91.44 m (s) EG: **Pre** 60 ± 2.5 **Post** 57 ± 2.5* CG: **Pre** 59 ± 2 **Post** 56 ± 1.5*
Sadowski et al. [41]	Regional *n* = 26 M EG, *n* = 14 14.0 ± 0.5 CG, *n* = 12 14.1 ± 0.5	Hydro-isokinetic ergometer (simulating underwater phase of fc)	6/3	EG: Hydro-isokinetic ergometer 6 sets of 50 s of work with 10 s rest CG: swim only	25 m fc	Isometric power, tethered SF, SL, SR	+ tethered SF	25 m (s) EG: **Pre** 14.71 ± 0.87 **Post** 14.53 ± 0.77 CG: **Pre** 16.89 ± 1.15 **Post** 16.78 ± 1.14
Sadowski et al. [21]	Regional *n* = 26 M EG, *n* = 12 15.8 ± 0.4 CG, *n* = 14 15.6 ± 0.6	Hydro-isokinetic ergometer (simulating underwater phase of fc)	12/3	EG: Hydro-isokinetic ergometer 30 s work/ 30 s rest × 10 with SR at 50–60 strokes/min CG: habitual hypertrophy training with focus on upper body strength	25 m fc	Isometric strength, tethered SF, SL, SR	+ isometric arm strength at 90º and 135º, SF in water and SV for both groups. Arm strength at 135º for EG	25 m (s) EG: **Pre** 13.51 ± 0.44 **Post** 12.95 ± 0.47* CG: **Pre** 13.89 ± 1.24 **Post** 13.51 ± 1.18*

Participant’s characteristics, method of resistance training, duration of training intervention and sessions per week, training program, swimming performance measured, positive effects of training intervention, findings in swim performance and whether they are significant (*p* < 0.05). EG = experimental training group and CG is control group

M = male, F = female, n = number, y = years old, yd = yards, fc = front crawl, SR = stroke rate, SL = stroke length, SF = swimming force, SV = swimming velocity

**Table 5:** Core training interventions

Reference	Competition level, sex. N intervention groups, age (y)	Non-specific dry-land resistance training	Week tr./ tr. per week	Resistance training program for EG and any changes in the CG habitual training	Performance measured	Other measured effects	Positive effects of training intervention	Findings in swim performance * Significant change from pre- to posttest (p < 0.05)
Karpinski et al. [49]	National *n* = 16 M EG, *n* = 8 20.2 ± 1.17 CG, *n* = 8 20.0 ± 1.9	Core training	6/3	EG: 40 s work/ 20 s rest × 4 with flutter kicks, single leg v-ups, prone physio ball trunk extension and Russian twists CG: swim only	50 m fc	Entry distance, entry velocity, time in air with take-off, dive angle, reaction time, time 5 m after turn, mean velocity after turn, SV, SR, SL and duration of 3 cycles	+ entry distance, reaction time, time 5 m after turning in both EG and CG, time in air with take-off, mean SV after turn and 50 m in EG	50 m (s) EG: **Pre** 25.24 ± 0.35 **Post** 24.94 ± 0.49* CG: **Pre** 26.82 ± 1.09 **Post** 26.64 ± 1.19
Sawdon-Bea and Benson [45]	Regional *n* = 32 16 M, 16 F EG, *n* = 16 15.0 ± 1.0 CG, *n* = 16 15.5 ± 1.5	Core training and shoulder flexibility	6/3	EG: 6 core exercises + 2 stretching exercises for 30 s work × 3 for all exercises CG: swim only	50 m fc	McGill trunk flexor test, pectoralis minor muscle length, posterior shoulder tightness, upper extremity strength	+ core strength	50 m (s) EG: **Pre** 31.52 ± 3.48 **Post** 32.06 ± 3.08 CG: **Pre** 31.69 ± 2.61 **Post** 31.74 ± 2.23
Weston et al. [44]	National *n* = 20 10 M, 10 F EG, *n* = 10 15.7 ± 1.2 CG, *n* = 10 16.7 ± 0.9	Core training	12/3	EG: Prone and side bridge: 30–120 s progression through intervention Leg raises and bird dog: 10–30reps Overhead SQ and Russian twist: 5–25 reps with 3–7 kg Shoulder press: 10–25reps CG: swim only	50 m fc	Shoulder extension strength, prone bridge test, EMG activity in core muscles	+ 50 m, shoulder extension strength, prone bridge, EMG activity in external obliquus, multifidus and latissimus dorsi	50 m (s) **Pre** 29.7 ± 2.1 **Post** 28.9 ± 0.40* CG: **Pre:** 28.0 ± 1.9 **Post** 27.8 ± 1.7

Participant’s characteristics, method of resistance training, duration of training intervention and sessions per week, training program, swimming performance measured, positive effects of training intervention, findings in swim performance and whether they are significant (*p* < 0.05). EG = experimental training group and CG is control group

M = male, F = female, n = number, y = years old, fc = front crawl, SV = swimming velocity

**Table 6:** Hypertrophy training interventions

References	Competition level, sex. N in intervention groups, age (y)	Non-specific dry-land resistance training	Week tr./ tr. per week	Resistance training program for EG and any changes in the CG habitual training	Performance measured	Other measured effects	Positive effects of training intervention	Findings in swim performance * Significant change from pre- to posttest (p < 0.05)
Junior et al. [48]	National *n* = 14 M EG, *n* = 7 15–16 CG, *n* = 7 15–16	Hypertrophy training	8/2	ST: 3 × 10 reps of BP, leg press, leg curl, triceps extensions at 60–80% 1RM CG: swim-only	50 m fc	RPE	+ 25 m fc	25 m (s) ST: **Pre** 13.12 ± 0.64 **Post** 12.85 ± 0.38* CG: **Pre** 13.15 ± 0.54 **Post** 13.13 ± 0.47 50 m (s) ST: **Pre** 27.52 ± 0.44 **Post** 27.14 ± 0.64 CG: **Pre** 27.55 ± 0.54 **Post** 27.51 ± 0.92
Lopes et al. [50]	National *n* = 20 14 M, 6 F EG, *n* = 11 20.45 ± 1.63 CG, *n* = 9 20.67 ± 2.00	Hypertrophy training	8/1	EG: 3–5 set × 6–12 reps of BP, SQ, CMJ, medical ball throw at 60–80% of 1RM CG: swim only	50 m, 100 m fc	SF, SL, mean SV, 1RM SQ and BP, max CMJ and medical ball throw	+ 100 m, 2nd 50 m in 100 m, SF in 2nd 50 m of 100 m, SI in 100 m and 1^st^ 50 m of 100 m 50 m, 1st 25 m of 50 m, SF in 50 m, BP	50 m (s) EG: **Pre** 29.65 ± 2.94 **Post** 28.47 ± 2.25* CG: **Pre** 31.70 ± 2.45 **Post** 31.61 ± 2.59 100 m (s) EG: **Pre** 67.04 ± 8.06 **Post** 64.13 ± 6.46* CG: **Pre** 71.08 ± 6.71 **Post** 71.78 ± 7.35
Tanaka et al. [47]	National *n* = 24 M EG, *n* = 12 19.17 ± 0.32 CG, *n* = 12 19.50 ± 0.26	Hypertrophy	8/3	EG: 8–12 reps × 3 of dips, chin-ups, lat pull-down, elbow extensions, bent arm flies CG: swim only	22.9 m fc	22.9 m sprint swimming, testosterone, cortisol, body fat, swim bench power, swimming power, SR, SL	+ none	22.9 m (s) EG: **Pre** 11.01 ± 0.15 **Post** 11.17 ± 0.18 CG: **Pre** 11.34 ± 0.23 **Post** 11.57 ± 0.09
Trappe and Pearson [8]	National *n* = 10 M EG, *n* = 5 20.1 ± 1.2 CG, *n* = 5 20.1 ± 1.2	Hypertrophy training	6/2	EG: 8–12 reps × 3 of weight assisted pull-ups and dips CG: habitual hypertrophy training. 8–12 reps × 3 of full-body exercises	22.9 m, 365.8 m (400yd) fc	Max. sprint swimming, swim bench power, swimming power, SR, SL	+ Max. sprint swimming (22.9 m), max. arm power in 1 of 3 methods in the swim- bench (WAG)	365.8 m (s) EG: **Pre** 255.15 ± 3.52 **Post** 245.41 ± 2.03* CG: **Pre** 255.28 ± 6.11 **Post** 245.50 ± 3.57*

Participant’s characteristics, method of resistance training, duration of training intervention and sessions per week, training program, swimming performance measured, positive effects of training intervention, findings in swim performance and whether they are significant (*p* < 0.05). EG = experimental training group and CG is control group

M = male, F = female, n = number, y = years old, yd = yards, fc = front crawl, RPE = ratings of perceived exertion with Borg’s scale, BP = bench press, SQ = squat, CMJ = countermovement jump, 1RM = 1 repetition maximum, SF = swimming force, SL = stroke length, SV = swimming velocity, SR = stroke rate, WAG = weight-assisted group

**Table 7:** Maximal strength training interventions

References	Competition level, sex. N intervention groups, age (y)	Non-specific dry-land resistance training	Week tr./ tr. per week	Resistance training program for EG and any changes in the CG habitual training	Performance measured	Other measured effects	Positive effects of training intervention	Findings in swim performance * Significant change from pre- to posttest (*p* < 0.05)
Girold et al. [12]	Regional to national *n* = 14 7 M, 7 F EG, *n* = 7 16.5 ± 2.5 CG, *n* = 7 16.5 ± 1.5	Maximal strength training	12/2	ST: 6 reps × 3, BP, pull ups, barbell draws, SQ, plyometrics CG: 45 min aerobic cycling	50 m fc	SL, SR, SD, elbow flexor and extensor strength	+ 50 m, SD, elbow extensor strength	50 m (s) ST: **Pre** 29.59 ± 2.88 **Post** 28.50 ± 2.00* CG: **Pre** 31.35 ± 2.30 **Post** 31.15 ± 1.85
Girold et al. [51]	National *n* = 16 8 M, 8 F EG, *n* = 8 21.1 ± 1.4 CG, *n* = 8 24.2 ± 4.6	Maximal strength training	4/3	ST: 6 reps × 3 pull ups, latissimus pull down, swim-bench latissimus pull-downs 80–90% 1RM CG: swim only	50 m fc	SL, SR, peak torque of arm extensors in isometric, arm extensors in concentric and arm extensors in eccentric	+ 50 m, SL, peak torque in concentric conditions	50 m (s) ST: **Pre** 26.84 ± 1.90 **Post** 26.30 ± 0.34* CG: **Pre** 28.57 ± 2.20 **Post** 28.43 ± 0.17
Schumann et al. [42]	National *n* = 16 10 M, 6 F EG, *n* = 9 14.8 ± 1.0 CG, *n* = 7 15.1 ± 1.1	Explosive and maximal strength training vs hypertrophic strength training	16/3	Wk 1–7: EG and CG trained 6–10 reps × 3 at 75–85% of 1RM of whole-body DLST Wk 8–16: EG trained 3–4reps × 4 at 85–90% of 1RM (SQ, deadlifts, BP, pull-ups) and explosive DLST (throws and jumps) CG: cont. with same training as in wk 1–7	400 m fc	1RM half-SQ and BP, CMJ, swim-start performance, RPE	+ 1RM half-SQ and BP, CMJ	400 m (s) EG: **Pre** 297 ± 17 **Post** 288 ± 11 CG: **Pre** 298.5 ± 13.5 **Post** 292.5 ± 8.5
Strass [43]	Regional *n* = 19 17 M, 2 F EG, *n* = 10 16.6 ± 1.2 CG, *n* = 9 17.8 ± 3.9	Maximal strength training	6/4	EG: Arm extensor muscle training at 1–3 sets of 1–3 reps at 90–100% of 1 RM CG: swim only	50 m fc	Isometric arm extension force, rate of force development, SR, SL	+ 50 m, 25 m, isometric arm extensor force, rate of force development, SR, SL	50 m (s) EG: **Pre** 28.25 ± 1.28 **Post** 27.62 ± 1.22* CG: no change, no values reported

Participant’s characteristics, method of resistance training, duration of training intervention and sessions per week, training program, swimming performance measured, positive effects of training intervention, findings in swim performance and whether they are significant (*p* < 0.05). EG = experimental training group and CG is control group

M = male, F = female, n = number, y = years old, fc = front crawl, BP = bench press, SQ = squat, CMJ = countermovement jump, DLST = dry-land strength training, 1RM = 1 repetition maximum, SF = swimming force, SL = stroke length, SV = swimming velocity, SR = stroke rate, SD = stroke depth, RPE = ratings of perceived exertion with Borg’s scale

**Table 8:** Plyometric training interventions

References	Competition level, sex. N intervention groups, age (y)	Non-specific dry-land resistance training	Week tr./ tr. per week	Resistance training program for EG and any changes in the CG habitual training	Performance measured	Other measured effects	Positive effects of training intervention	Findings in swim performance * Significant change from pre- to posttest (p < 0.05)
Potdevin et al. [46]	Regional *n* = 23 10 M, 13 F EG, *n* = 12 14.3 ± 0.2 CG, *n* = 11 14.1 ± 0.2	Plyometric training	6/2	EG: Unloaded plyometric jump training (long, lateral and depth) Total jumps: 220 first week with linear increasing up to 498 CG: swim only	50 m, 400 m fc	CMJ, SJ, gliding test (max. speed and mean acceleration), 25 m front crawl without dive, 25 m kicking	+ CMJ, SJ, max. glide speed, 50 and 400 m front crawl. Mean acceleration during gliding for both groups	50 m (s) EG: **Pre** 40.00 ± 5.88 **Post** 38.76 ± 4.57* CG: **Pre** 40.98 ± 4.77 **Post** 40.32 ± 3.61 400 m (s) EG: **Pre** 434.78 ± 47.82 **Post** 416.67 ± 39.41* CG: **Pre** 454.55 ± 41.67 **Post** 449.44 ± 30.44*

Participant’s characteristics, method of resistance training, duration of training intervention and sessions per week, training program, swimming performance measured, positive effects of training intervention, findings in swim performance and whether they are significant (*p* < 0.05). EG = experimental training group and CG is control group

M = male, F = female, n = number, y = years old, fc = front crawl, CMJ = countermovement jump, SJ = squat jump

**Table 9:** A combined strength and endurance training intervention

Reference	Competition level, sex. N intervention groups, age (y)	Non-specific dry-land resistance training	Week tr./ tr. per week	Resistance training program for EG and any changes in the CG habitual training	Performance measured	Other measured effects	Positive effects of training intervention	Findings in swim performance * Significant change from pre- to posttest (p < 0.05)
Aspenes et al. [9]	National *n* = 20 8 M, 12 F EG, *n* = 11 17.5 ± 2.9 CG, *n* = 9 15.9 ± 1.1	Combined strength and endurance training	11/2	EG: Strength: 5 reps × 3 latissimus pull down at 60–75% 1RM Swimming: 4 × 4 min intervals high intensity CG: swim only	50 m, 100 m, 400 m freestyle	Land strength, SF, VO_2peak_, cost of swimming, SR, SL and max. SV	+ 400 m, land strength, swimming force	50 m (s) EG: **Pre** 28.88 ± 2.00 **Post** 28.55 ± 1.80 CG: **Pre** 29.35 ± 1.71 **Post** 29.16 ± 1.76 100 m (s) EG: **Pre** 63.00 ± 4.12 **Post** 62.05 ± 3.82 CG: **Pre** 64.08 ± 4.18 **Post** 64.06 ± 4.80 400 m (s) EG: **Pre** 290.43 ± 16.26 **Post** 286.43 ± 16.64* CG: **Pre** 290.08 ± 16.20 **Post** 290.40 ± 18.24

Participant’s characteristics, method of resistance training, duration of training intervention and sessions per week, training program, swimming performance measured, positive effects of training intervention, findings in swim performance and whether they are significant (*p* < 0.05). EG = experimental training group and CG is control group

M = male, F = female, n = number, y = years old, fc = front crawl, 1RM = 1 repetition maximum, SF = swimming force, SR = stroke rate, SL = stroke length, SV = swimming velocity

For the in-water arm strength training groups, the collective mean improvement was 1.7% (Table 10). The smallest improvement was 0% [33] and the largest improvement was 3.2% [38]. The in-water training interventions with added resistance had a 2.5 ± 1.9% mean performance improvement. There was only one specific in-water leg training intervention so there is not a collective mean, but the percent change for the one study was only 0.65% and not significant. For the swim-like dry-land resistance training groups, the mean improvement was 2.6 ± 1.9%. Lastly, we had non-specific dry-land strength training interventions. They were organized into subgroups. There was only one available plyometric training intervention and one intervention that combined endurance and strength training, so the mean improvement was based on the mean of the different swimming distances that the studies investigated. Collectively, the mean improvements of the plyometric trained group were 3.6 ± 0.8%. In the combined endurance and strength training group, the mean was 1.3 ± 0.2%. The core training interventions (1.9% improvement), hypertrophy training interventions (2.6% improvement) and maximal strength training interventions (2.7% improvement) all involved several studies. All the non-specific dry-land interventions had a collective mean change in performance of 2.5 ± 1.5%.

**Table 10:** An overview of the collective mean ± SD for each of the different types of training interventions

Type of training intervention	Mean ± SD (% improvement)
Specific in-water arm strength training	1.7 ± 1.2
Specific in-water training with added resistance	2.5 ± 1.9
Specific in-water leg strength training	0.65
Dry-land swim-like resistance training	2.6 ± 1.9
Non-specific dry-land core training	1.9 ± 0.8
Dry-land hypertrophy training	2.6 ± 1.9
Dry-land maximal strength training	2.7 ± 0.8
Non-specific dry-land plyometric training	3.6 ± 0.8
Combined strength and endurance training	1.3 ± 0.2

**Fig. 3:**
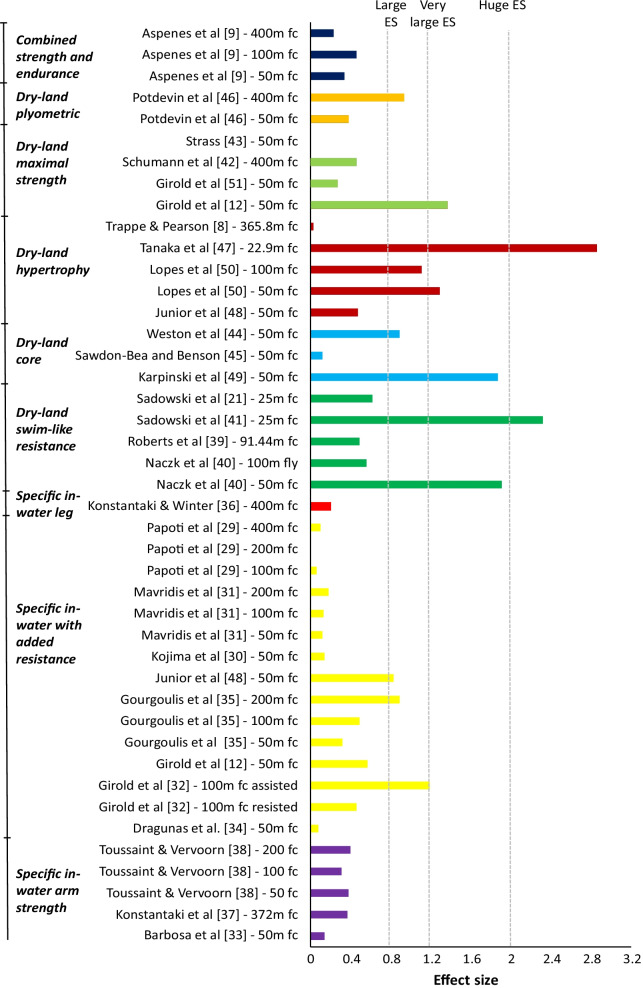
Effect sizes (ESs) between the control and experimental groups

Most of the interventions did not reach medium ES. Three studies showed a medium ES between groups [12, 21, 40], while six studies revealed large ES [32, 35, 44, 46, 48, 50] for the 100 m front crawl. Four studies showed very large ESs [12, 40, 49, 50], while only two studies showed huge ESs [41, 47] (Fig. 3).

## Discussion

The main objectives of this review were to examine previous literature on (1) how different approaches to strength training for competitive swimmers can improve swimming performance and (2) which form of strength training resulted in the largest improvement in swimming performance. Collectively, almost all the experimental groups, and some of the control groups, showed a decrease in total swimming time and thereby gained a positive outcome of the training intervention. The results varied from a 7.5% performance increase [35] to a −1.45% performance decrease [47], with an average increase of 2.2% in the specific in-water training group, 2.5% in the non-specific dry-land strength training group and 2.6% in the dry-land swim-like training group. Furthermore, most of the studies were done in relation to the performance of the front crawl.

### Method-Related Considerations

When assessing the results, there are important method-related inconsistencies that need to be considered. Firstly, there is a large age gap between the participants in the studies (13–24 years old), which leads to differences in competitive levels and training experiences that will influence the results. The highly skilled, older athlete with longer training experience has a smaller range of improvement than the younger more inexperienced athlete. Men were among the majority in the training groups (66.7%), and there was mixing of sexes in several of the groups. Some of the studies only had male participants [8, 21, 36, 37, 39, 41, 47–49]). Gourgoulis et al. [35] had young female participants and the rest of the studies had both male and female participants. Participants’ numbers ranged from 10 [8] to 82 [31], with an average of around 16 participants. Statistically, a low number of participants reduce the statistical impact of the study, and the value of the study’s findings must be evaluated accordingly.

Furthermore, there was a wide span in the duration of the training interventions. The shortest intervention lasted for 3 weeks [32] and the longest for 16 weeks [42], with an average of 8 weeks. This is problematic in the sense that the participants in the longer interventions had more time to adapt to the training, which could result in a more accurate representation of the effect that type of strength training had on swimming performance.

Another inconsistency is the three studies that did not apply a swim-only approach to their control groups [8, 21, 42]. These control groups performed their usual dry-land hypertrophy training, while their experimental groups performed dry-land swim-like strength training [21], maximal strength training [42] and weight-assisted hypertrophy training [8]. This makes it difficult to determine the effect of the training intervention as compared to that of a control group.

### In-water Specific Resistance Training

### Specific In-water Arm Strength Training

The interventions in this group were designed to increase arm strength through specific strength training in the water, and there were three eligible interventions. There were a hand paddle intervention [33], an arms-only intervention [37] and a POP device intervention [38]. It is difficult to conclude that this type of training has a definite positive or negative effect on swimming performance. Firstly, there is limited available research, since there are only three studies in this category. The mean of the three arm-strength interventions showed an improvement of 1.7 ± 1.2% (Table 10). However, Barbosa et al. [33] did not find a significant effect for their experimental group in a 50 m fc with 0% change in performance and a trivial change (0.14) between-group ES. This study was conducted over the span of only 4 weeks. This allows very little time for adaption to training and could explain the lack of results. Konstantaki et al. [37] also showed no significant improvement pre- and posttest in 372 m fc and a small improvement between-group ES. In this intervention, the EG performed 20% of the weekly swimming training with arms-only. The lack of improvement could be due to the fact that this form of training alone is not enough to gain more strength in the arms than normal swimming does. Although swimming performance did not improve, a 186 m arms-only trial did. This supports the principle of specificity. The EG improved the parameter they practiced, but there were transfer issues to swimming performance. Toussaint and Vervoorn [38] conducted tests on 50 m, 100 m and 200 m fc, whereas the experimental group showed a significant gain in all distances. The CG also showed gains in performance but only in the 100 m test. The ES was small. The device used in this intervention is highly specific to swimming and could be the reason that the EG improved their swimming performance. The CG performed the same sprint training as the EG but only showed a gain in the 100 m test, which could indicate that the chosen method of sprint training is effective, but the sprint training with the device was even more effective.

### Specific In-water Resistance Training

In this group of training interventions, the focus is specific in-water training with added resistance. This is a swim-specific way to gain strength and follows the principle of specificity that specifies that training should be as close as possible to the actual sport performance. The resistance is applied to the swimmers through resistance bands, parachutes or drag suits. The mean percentage for this group was 2.5 ± 1.9% (Table 10), and all studies, except Papoti et al. [29], had a positive effect on swimming performance. This tells us that this method is likely to result in a positive gain in swimming performance. A 2.5% change in performance is a considerable improvement in competitive swimming, but the SD shows that the variation of improvement differs greatly between the swimmers.

Assessing the drag suit and parachute trained experimental groups’ performances, there are large differences in results, despite the fact that these training methods arguably are very similar. In Dragunas et al. [34], the swimmers pulled a parachute behind them, and in Gourgoulis et al. [35], they wore a belt around their waist with pockets that filled with water when the swimmers swam, increasing the resistance. Dragunas et al. [34] had a 0.3% gain in 50 m fc performance, while Gourgoulis et al. [35] experienced a 3.2%, 5.1% and 7.5% gain in 50 m, 100 m and 200 m tests, respectively. The between-group ES was trivial in Dragunas et al. [34], and in the 50 m, 100 m and 200 m tests in Gourgoulis et al. [35], it was small to large (0.32, 0.49 and 0.89, respectively). The large variance in results could be due to the fact that the swimmers in Dragunas et al. [34] were 19–20 years old, and in Gourgoulis et al. [35], the swimmers were only girls that were 13–14 years old. The younger athletes have a large potential for improvement and possibly have greater use of this form of strength training than the older athletes that are already much stronger. Furthermore, the Gourgoulis et al. [35] intervention lasted for 11 weeks, where as Dragunas et al. [34] intervention lasted for only 5 weeks. The 11-week intervention allows for more time for adaption to training and could explain some of the reasons that this intervention had better results than the 5-week intervention.

For the resistance band trained experimental groups, the results were more consistent. In the resistance band trained groups, there were two methods of using the resistance band. Most studies had the participants swim out with the band to give resistance [29–31, 48]. The age of the participants ranged from 14 to 16 years old in all studies, and the mean gain in performance for the four interventions was about 2.0%. One study had a combined resisted-assisted method where the swimmers swam resisted one way and assisted the other way [12]. This resulted in a 3.0% gain in performance. Girold et al. [32] had two experimental groups, one group swam resisted, and one group swam assisted, and then compared the two. The resisted group had a 2.0% gain in performance, which correlated with the other four resisted trained groups, while the assisted group had a 0.9% gain in performance and the lowest gain in performance for all the resistance band trained groups. These results indicate that if training with a resistance band is desired, a combined resisted-assisted method might be most successful. However, only one study had this approach, which makes the results tentative.

### Specific In-water Leg Training

The arms are generally considered the main propulsive factor in swimming and are, therefore, often the focus when discussing strength training in swimming, even though the legs contain large muscles with great strength potential. Aspenes and Karlsen [1] speculate the legs in swimming are more of a stabilization factor to reduce drag rather than increase propulsion and swimming velocity. Gullstrand and Holmer [55] performed a correlation study with international level swimmers over a 5-year period and found that tethered leg kicking was not related to swimming performance. On the other hand, Schumann and Rønnestad [56] mentioned that a gain in leg strength could result in improvement in start and turn performance, which could result in an all-over gain in swimming performance. Only one study was eligible for this review. Konstantaki and Winter [36] executed a leg kicking study but found no significant change in a 400 m fc (-0.65%). The between-group ES was small (0.2). Arguably, a 0.65% gain in performance for an experienced swimmer is a positive effect, but considering the distance swam (400 m fc), this result is not of any real practical importance. Due to the limited availability of research, it was not possible to draw a definite conclusion of how an in-water leg training intervention could affect swimming performance. Compared to the in-water arm-strength training and the in-water resistance training, it seemingly would be beneficial to perform these methods of resistance training over the in-water leg training.

### Dry-Land Swim-Like Resistance Training

This form of strength training is considered the most specific to swimming, when on dry land. It mimics the swimming performance, but it lacks specificity in the sense that the arms are isolated, the drag phase is longer than a swimming stroke in the water, and the distribution of the drag forces at various joint angles is not like in-water swimming [57]. It is also worth considering that this form of training demands specialized equipment that may not be as accessible as a swimming pool, rubber bands or a strength training room.

The collective mean for these intervention groups was a 2.6 ± 1.9% enhancement in performance, but there were large differences in performance changes. The greatest change was in the Roberts et al. [39] study on 91.44 m fc, with a 5.0% increase in performance. However, this is probably not due to the swim bench training, as the CG also experienced large and almost the same gain in performance (5.1%) over the 10-week intervention. This could mean that other substantial factors have impacted the swimmers, as a 5% improvement is a huge enhancement in 91.44 m. Roberts et al. [39] speculated whether the improvements could be due to the fact that earlier in the season the main goal was to improve the biomechanics of the stroke and maximal VO_2,_ while in the second part of the season, when the intervention took place, the focus shifted to a more high stroke turn over, anaerobic power and endurance, which are all important factors in a 91.44 m performance. The shift in focus obviously had a positive impact on the swimmer’s performance, but it is not certain that the swim bench training had an extra positive effect compared to the CG. Naczk et al. [40] used the same swim bench method as Roberts et al. [39] but found significant changes in the 50 m fc and 100 m butterfly (0.79% and 1.83%, respectively) in the EG only. However, Naczk et al. [40] also had limitations, as the duration of the intervention was relatively short (4 weeks). This provided little time to adapt to the training, making the findings uncertain. Naczk et al. [40] believed that some of the effects could be explained on the basis of placebo.

Sadowski et al. [41] and Sadowski et al. [21] used a device similar to the swim bench called a hydro-isokinetic ergometer. Sadowski et al. [41] performed a 6-week intervention and found a nonsignificant 1.2% gain in performance in the EG, while Sadowski et al. [21] performed a 12-week intervention and the EG had a significant 4.1% change in performance (as did the CG) (2.7%). The control group did not perform a swim-only method, but rather dry-land hypertrophy training. This made it difficult to ascertain the true effect of the ergometer vs. normal swimming practice, but it made it possible to compare swim-specific dry-land training and non-specific strength training. Both methods resulted in significant gains in performance, but the swim-specific method had greater improvements than traditional strength training. When comparing the two ergometer trained experimental groups, Sadowski et al. [21] showed the largest performance enhancement compared to Sadowski et al. [41], which was probably due to the duration of the interventions (12 weeks vs. 6 weeks).

### Dry-land Non-specific Resistance Training

### Core Training

This type of training is non-specific to swimming, but it is widely used by swimmers due to the unstable nature of water, which demands a strong core for a purposively forward propulsion. The collective mean change in this group was 1.9 ± 0.8%, all measured in the 50 m fc (Table 10), which is a substantial improvement in such a short distance for experienced swimmers. However, Sawdon-Bea and Benson [45] indicated an insignificant change in performance for the EG of 1.7%, which was hard to explain. Some possible reasoning for the absence of a significant increase in performance probably lies in the fact that the participants were only experienced high school swimmers competing at a regional level, which could have affected the quality of core training they received due to variations in levels between the participants at this level. Furthermore, Sawdon-Bea and Benson [45] did not specify what kind of core exercises the participants executed. The exercises could lack an element of specificity that the other interventions had and therefore, was not always transferred to the swimming performance for each participant.

### Traditional Resistance Training

Traditional resistance training is widely used in swimming and involves conventional gym-based strength training. In this review, traditional resistance training was divided into hypertrophy training, maximal strength training, plyometric training and a combined endurance and strength training regimen. The mean change in performance for these methods was 2.6 ± 1.5%, with only one study reporting a negative outcome in swimming performance [47]. This was a hypertrophy training intervention with a focus on upper body strength. The EG in a study by Tanaka et al. [47] increased their weights by 25–35% over the span of the intervention but showed no gain in swimming performance or swim bench power. The lack of positive transfer could be due to a lack of specificity in the training. This may be an insufficient explanation for the decrease in performance, while the mean gain in performance in the hypertrophy trained groups was 2.6%. Trappe and Pearson [8] applied a weight-assisted hypertrophy strength training program for the EG, while the CG performed free-weight hypertrophy training. This made it problematic to investigate the differences between a combined hypertrophy and swimming training regimen and swimming training alone. Both the weight-assisted group and free-weight group gained significant change in the 365.8 m fc (around 3.8% for both groups) and had a trivial (0.03) between-group ES, which tells us that there is little difference between the two training methods.

It does not appear to be of importance whether the hypertrophy training was full body or upper body focused, as similar improvements were found after performing a full body strength training routine rather than an upper body focused one [21, 42, 48, 50]. This strays from the principle of specificity that says the upper body is the primary propulsion factor in swimming and that it seemingly would be most beneficial to perform upper body strength training. However, this is in line with the in-water resistance training groups where the added resistance trained group gained larger performance enhancements than the in-water arm strength only training group. This could mean that a full body focused resistance training regimen, regardless of whether it is in-water or on dry-land, is more beneficial to the transfer to swimming performance rather than just focusing on one part of the body (e.g., the arms).

In the maximal strength training interventions, the collective mean was 2.7 ± 0.8%, which states a possible likelihood of change in performance. Most studies conducted only the maximal strength training intervention and compared it with a control group, which gives a clear indication if the strength training has a positive effect or not. Only Aspenes et al. [9] conducted a study where they combined a 4 × 4 min endurance program and maximal strength training (a pull-down exercise designed to mimic the butterfly stroke). They investigated the 50 m, 100 m and 400 m freestyle, and the mean change in performance in the three distances was 1.3%. The only significant changes were found in the 400 m performance. The between-group ES never reached a significant level, except in the 100 m performance, with a small between-group ES (0.46). Therefore, in this study, it is difficult to predict whether the gain in the 400 m performance is due to the maximal strength training or to the endurance training, but it is suggested to be related to the strength portion of the program since the VO_2max_ and work economy remained unchanged [9]. Aspenes et al. [9] was the only study that tried to apply a specificity aspect to maximal strength training. This seemingly did not make a difference in the swimming performance, as the other maximal strength training groups had larger improvements in performance (2–3%). This may indicate that a general increase in strength is sufficient and preferred for an improved swimming performance.

Only one study investigated the effect of plyometric training on total swimming performance [46]. Plyometric studies in swimming are often related to start-and-turn performance and Bishop et al. [54] showed positive effects in swimming performance after this kind of training. Potdevin et al. [46] showed a 3.1% and 4.7% change in the 50 m and 400 m fc, respectively, which is a considerable improvement. The CG also significantly improved their 400 m performance (1.1%), which makes it unclear if it is the strength training intervention or other factors that influenced the swimmer’s performance. Nevertheless, the gain in performance was larger in the EG, which tells us that maybe plyometric training had a positive effect. In the 50 m performance, only the EG improved their performance. This could be due to the shorter distance, where start performance plays a greater role in total performance than in the 400 m, and plyometrics has been shown to positively affect start performance [54]. However, one study is not enough to conclude whether plyometric dry-land training has a positive or negative effect on swimming performance.

### Comparison of Training Methods

It is an established fact that specificity in training is necessary for positive transfer to performance, but it is curious to note that all three groups had a mean gain in performance of 2–3%, which is a considerable improvement for competitive swimmers, regardless of what kind of strength training they performed. Regarding mean gain in performance, specific in-water training methods had a 2.2% mean gain, dry-land swim-like resistance training had a 2.6% mean gain, and dry-land non-specific strength training had a 2.4% mean gain. Thereby, the current literature demonstrates that various resistance training methods can positively impact swimming performance.

Dry-land swim-like resistance training showed the greatest change in performance, but this is also the group with the fewest studies and participants. Only one of four studies showed a statistically significant change in performance, which could be due to the lack of specificity in the movement of the swim bench. The non-specific dry-land training methods were used in 13 different studies. Three subgroups contained several interventions and made it possible to draw the following conclusions: (1) core training showed a 1.9% gain in performance, (2) hypertrophy training a 2.6% gain and (3) maximal strength training a 2.7% gain, which showed that all methods could positively affect swimming performance. Core training could be beneficial due to the nature of swimming, but it needs to be specific in the way that the core training on land is transferable to in-water swimming. Both hypertrophy and maximal strength training led to similar and considerable gains in swimming performance, which indicates that gain in muscle strength, even though the training is not specific to swimming, is transferable to swimming and has positive effects on performance. These methods showed substantially larger effects than core training, which might predict that hypertrophy or maximal strength training could be more useful to the swimmer than core training alone. Specific in-water training with 12 included studies had the least gain in performance. Nevertheless, the results showed that specific in-water strength training also leads to a probable gain in performance. The greatest all-over individual swimming performance improvements were found in this group. Within this group, the interventions with added resistance had greater gains in performance compared to the arms and legs focused interventions, which could be due to the principle of specificity. The act of swimming with a rubber band is more specific to swimming than swimming only using the arms.

When discussing the principle of specificity, it would be reasonable to conclude that the specific in-water training should lead to a greater gain in performance. There could be several reasons for this outcome, and due to the limited availability of literature, it is hard to make a definite conclusion. One reason may be that dry-land hypertrophy and maximal strength training leads to greater improvement in muscle strength than in-water resistance training and that might be what is needed to significantly increase swimming performance. It has been shown that younger athletes benefit from in-water resistance training [30, 31, 35], but for stronger and more experienced swimmers, in-water resistance does not necessarily result in increased muscle strength, which could be why dry-land strength training is more effective for improvement in swimming performance.

This review has three limitations. First, as there are limited studies in some of the categories it is still not possible to provide a definitive statement about which resistance training method is the most effective one to increase swimming performance. Secondly, it is possible that some studies were not found in the search process. Lastly, there are many other factors that could influence swimming performance over time which are possible confounding variables outside of the intervention programs since training is a multifactorial process.

## Conclusion

The main finding of the review was that all three main training method groups had interventions that led to significant gains in front crawl swimming performance. While the change in performance ranged from −1.45 to 7.5%, the majority of the interventions led to a 2–3% gain in performance. It seems that dry-land swim-like resistance training, hypertrophy training and maximal strength training are the most successful strength training methods to increase swimming performance, especially for more experienced and stronger senior competitive swimmers. Thus, for coaches and swimmers, we suggest including these training methods in the training regime. However, the findings did not follow the principle of specificity that specific in-water strength training is more beneficial to swimming performance than non-specific resistance training. It must not be construed that dry-land strength training can replace specific swimming training, but it might be a positive addition to the training program. It is clear that any of the different resistance training methods led to greater gains in swimming performance compared to the control groups where the subjects had a swim-only approach to training. Further research with high-quality randomized controlled trials and longer training interventions with full documentation of all training plans using elite senior swimmers are necessary to accurately interpret the results of the various forms of strength training and to provide guidelines for resistance training for swimmers.

## Data Availability

Please contact author for data requests.

## Abbreviations

1RM: 1 Repetition maximum
BP: Bench press
CG: Control group
CMJ: Countermovement jump
DLST: Dry-land strength training
EG: Experimental group
ES: Effect size
F: Female
fc: Front crawl
M: Male
n: Number
RPE: Ratings of perceived exertion with Borg’s scale
SF: Swimming force
SL: Stroke length
SQ: Squat
SR: Stroke rate
SV: Swimming velocity
VO_2_: Oxygen uptake
VO_2max_: Maximal oxygen uptake
VT: Ventilatory threshold
WAG: Weight-assisted group
y: Years old
